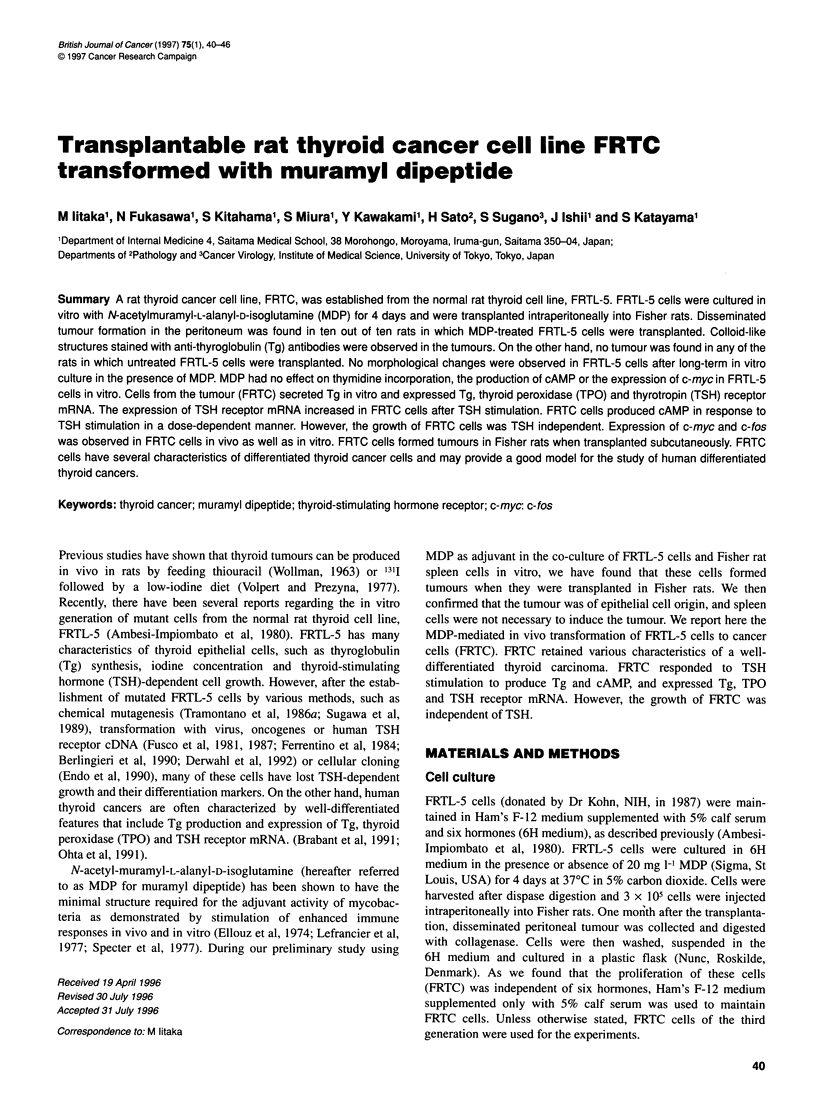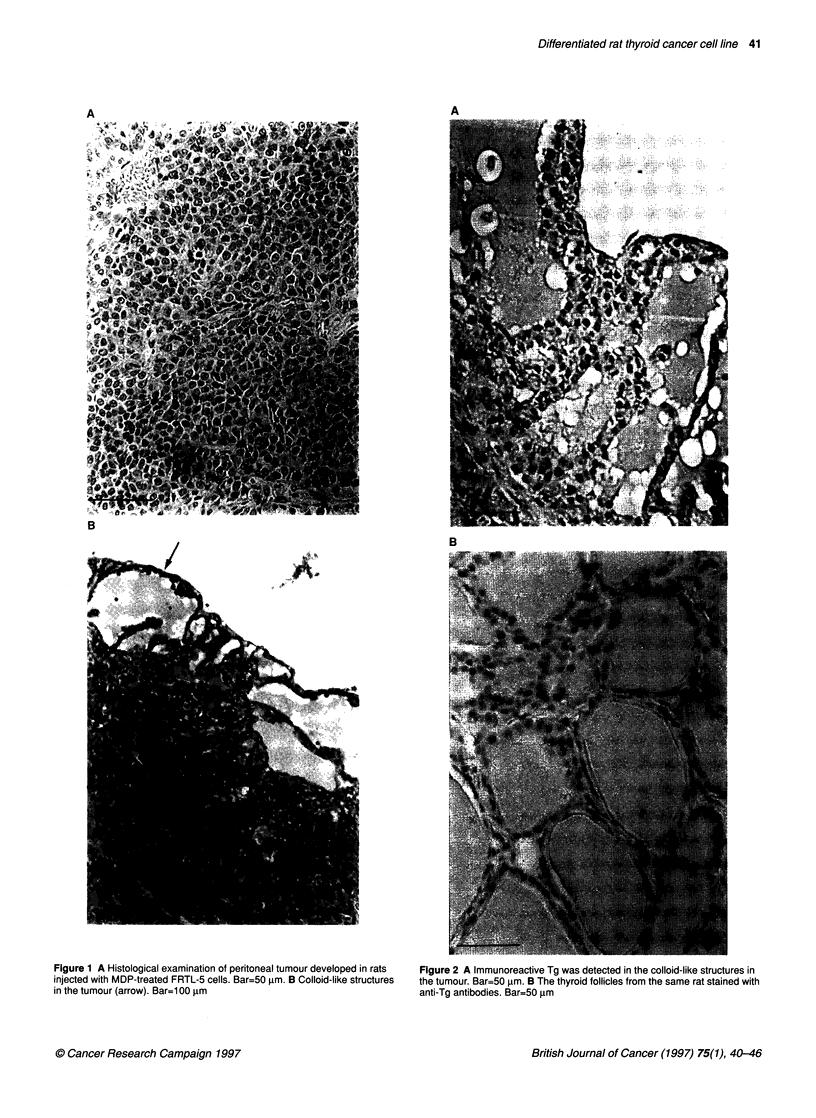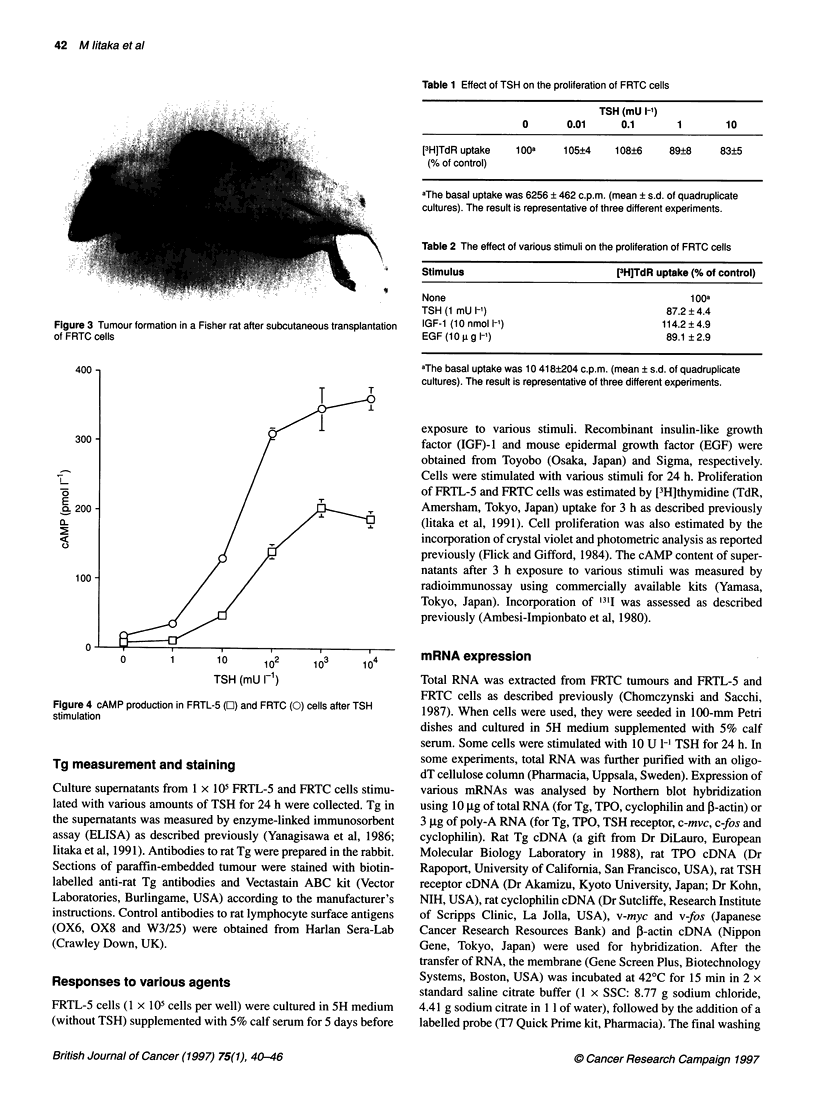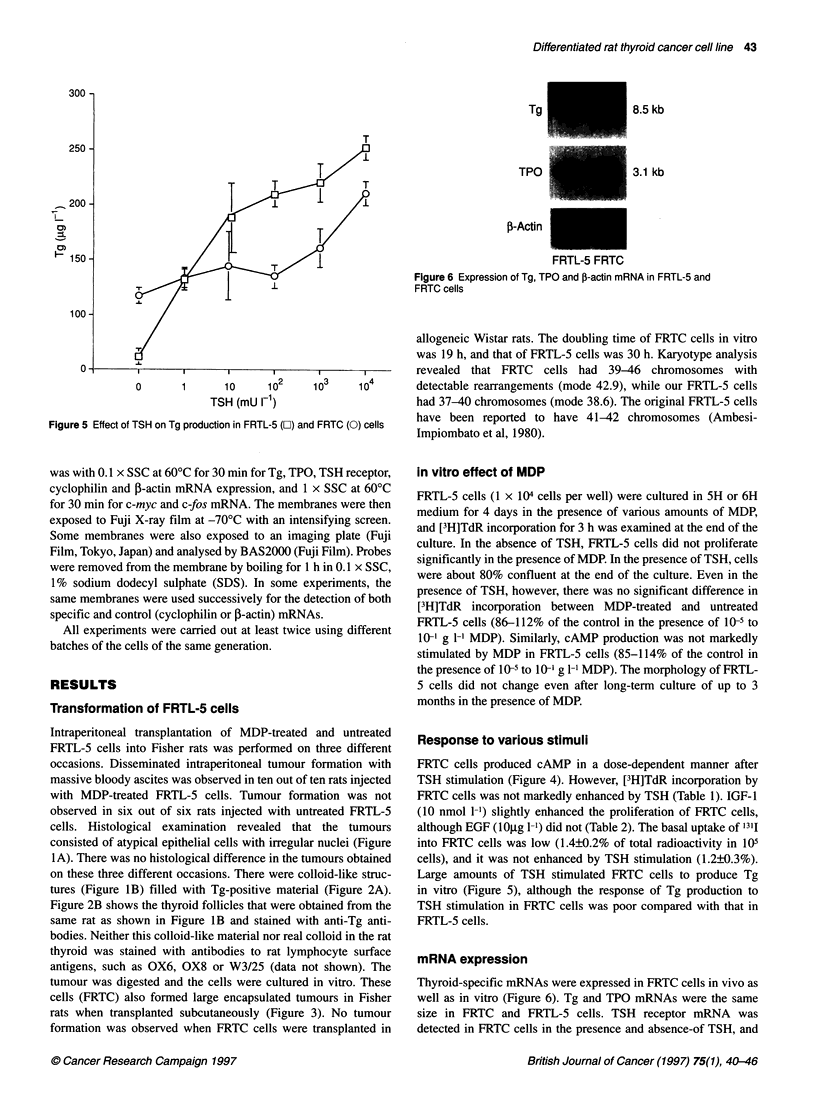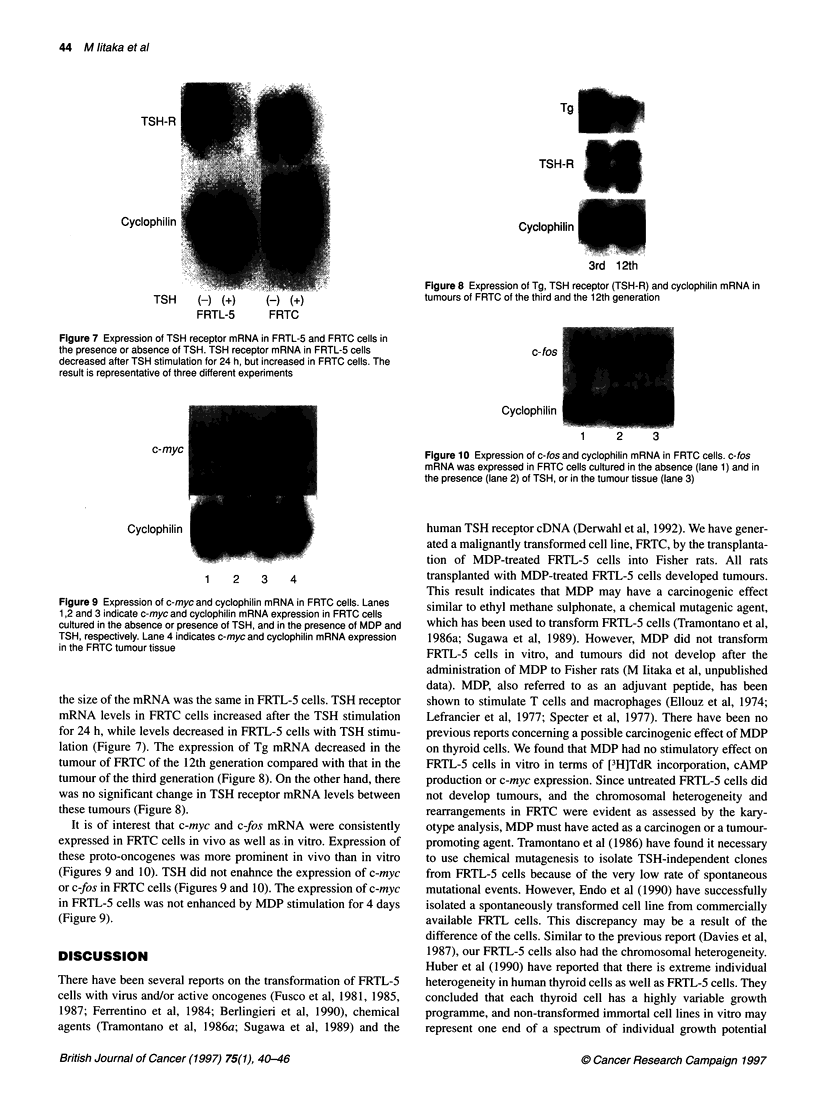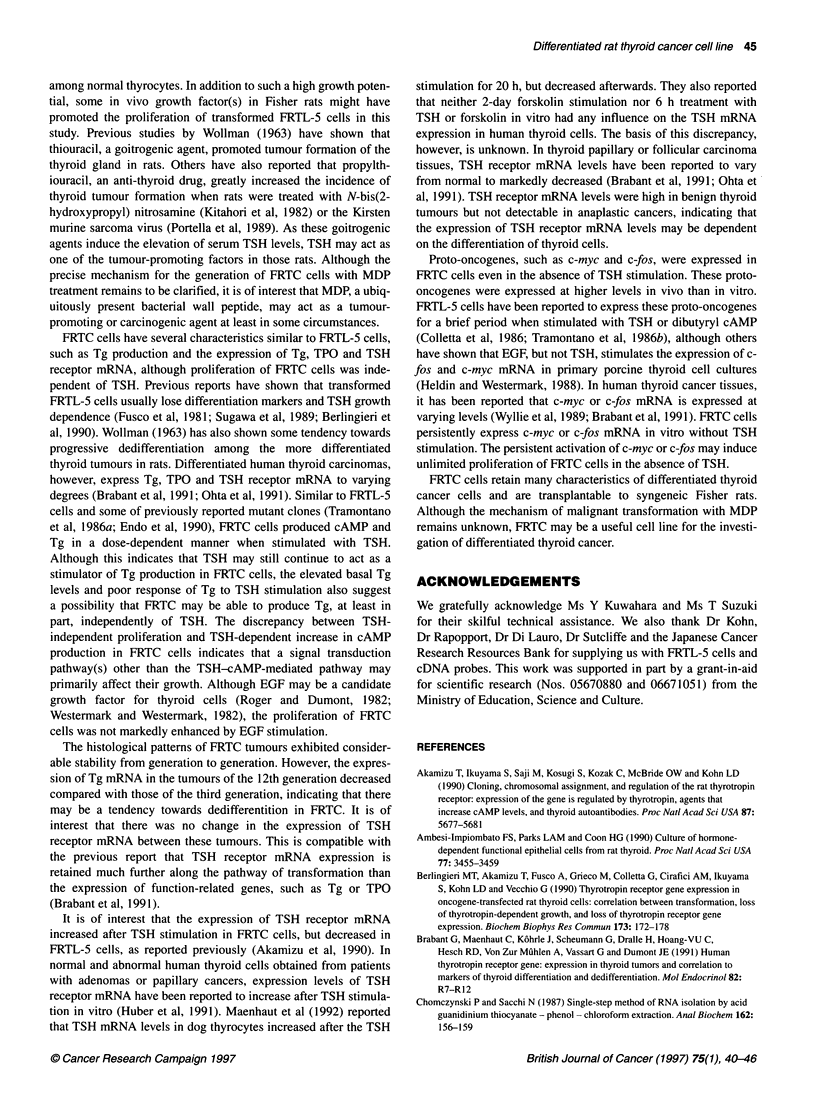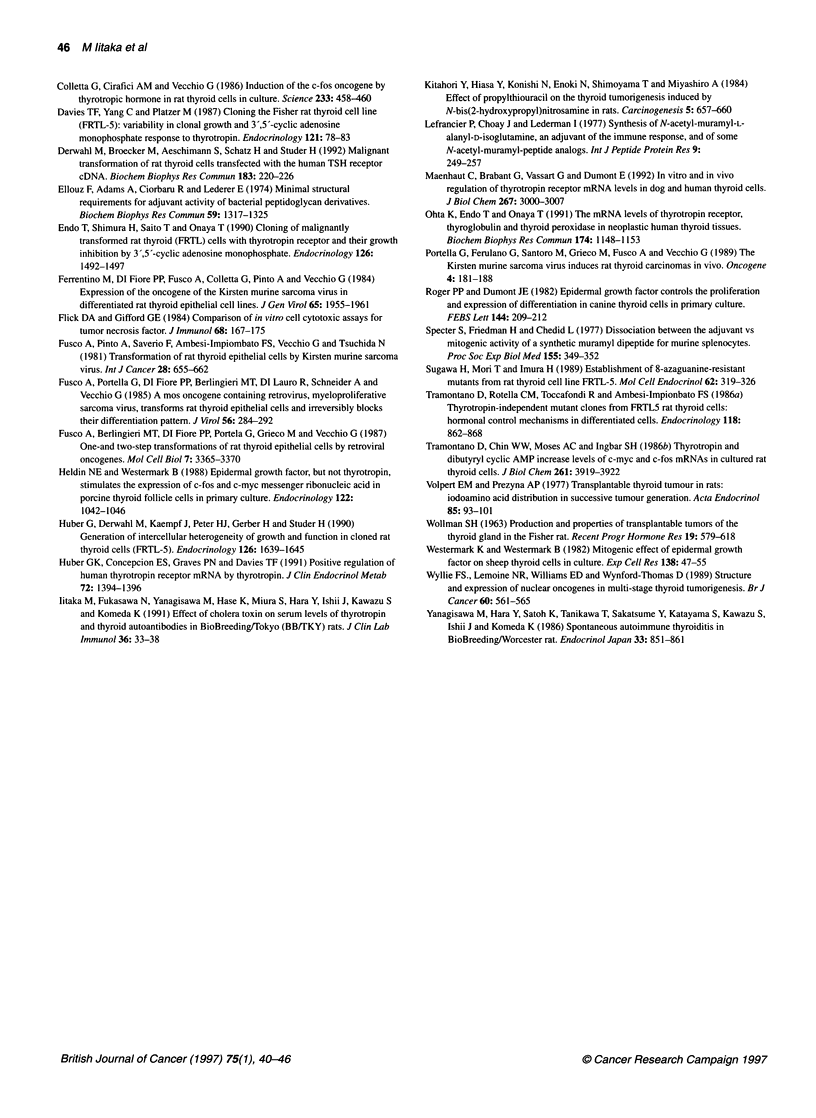# Transplantable rat thyroid cancer cell line FRTC transformed with muramyl dipeptide.

**DOI:** 10.1038/bjc.1997.7

**Published:** 1997

**Authors:** M. Iitaka, N. Fukasawa, S. Kitahama, S. Miura, Y. Kawakami, H. Sato, S. Sugano, J. Ishii, S. Katayama

**Affiliations:** Department of Internal Medicine 4, Saitama Medical School, Japan.

## Abstract

**Images:**


British Journal of Cancer (1997) 75(1), 40–46
© 1997 Cancer Research Campaign

### Transplantable rat thyroid cancer cell line FRTC transformed with muramyl dipeptide

**M Iitaka[1], N Fukasawa[1], S Kitahama[1], S Miura[1], Y Kawakami[1], H Sato[2], S Sugano[3], J Ishii[1] and S Katayama[1]**

[1]Department of Internal Medicine 4, Saitama Medical School, 38 Morohongo, Moroyama, Iruma-gun, Saitama 350–04, Japan;
Departments of [2]Pathology and [3]Cancer Virology, Institute of Medical Science, University of Tokyo, Tokyo, Japan

**Summary** A rat thyroid cancer cell line, FRTC, was established from the normal rat thyroid cell line, FRTL-5. FRTL-5 cells were cultured in vitro with *N*-acetylmuramyl-L-alanyl-D-isoglutamine (MDP) for 4 days and were transplanted intraperitoneally into Fisher rats. Disseminated tumour formation in the peritoneum was found in ten out of ten rats in which MDP-treated FRTL-5 cells were transplanted. Colloid-like structures stained with anti-thyroglobulin (Tg) antibodies were observed in the tumours. On the other hand, no tumour was found in any of the rats in which untreated FRTL-5 cells were transplanted. No morphological changes were observed in FRTL-5 cells after long-term in vitro culture in the presence of MDP. MDP had no effect on thymidine incorporation, the production of cAMP or the expression of *c-myc* in FRTL-5 cells in vitro. Cells from the tumour (FRTC) secreted Tg in vitro and expressed Tg, thyroid peroxidase (TPO) and thyrotropin (TSH) receptor mRNA. The expression of TSH receptor mRNA increased in FRTC cells after TSH stimulation. FRTC cells produced cAMP in response to TSH stimulation in a dose-dependent manner. However, the growth of FRTC cells was TSH independent. Expression of *c-myc* and *c-fos* was observed in FRTC cells in vivo as well as in vitro. FRTC cells formed tumours in Fisher rats when transplanted subcutaneously. FRTC cells have several characteristics of differentiated thyroid cancer cells and may provide a good model for the study of human differentiated thyroid cancers.

**Keywords:** thyroid cancer; muramyl dipeptide; thyroid-stimulating hormone receptor; *c-myc*: *c-fos*

Previous studies have shown that thyroid tumours can be produced in vivo in rats by feeding thiouracil (Wollman, 1963) or $^{131}I$ followed by a low-iodine diet (Volpert and Prezyna, 1977). Recently, there have been several reports regarding the in vitro generation of mutant cells from the normal rat thyroid cell line, FRTL-5 (Ambesi-Impiombato et al, 1980). FRTL-5 has many characteristics of thyroid epithelial cells, such as thyroglobulin (Tg) synthesis, iodine concentration and thyroid-stimulating hormone (TSH)-dependent cell growth. However, after the establishment of mutated FRTL-5 cells by various methods, such as chemical mutagenesis (Tramontano et al, 1986*a*; Sugawa et al, 1989), transformation with virus, oncogenes or human TSH receptor cDNA (Fusco et al, 1981, 1987; Ferrentino et al, 1984; Berlingieri et al, 1990; Derwahl et al, 1992) or cellular cloning (Endo et al, 1990), many of these cells have lost TSH-dependent growth and their differentiation markers. On the other hand, human thyroid cancers are often characterized by well-differentiated features that include Tg production and expression of Tg, thyroid peroxidase (TPO) and TSH receptor mRNA. (Brabant et al, 1991; Ohta et al, 1991).

*N*-acetyl-muramyl-L-alanyl-D-isoglutamine (hereafter referred to as MDP for muramyl dipeptide) has been shown to have the minimal structure required for the adjuvant activity of mycobacteria as demonstrated by stimulation of enhanced immune responses in vivo and in vitro (Ellouz et al, 1974; Lefrancier et al, 1977; Specter et al, 1977). During our preliminary study using MDP as adjuvant in the co-culture of FRTL-5 cells and Fisher rat spleen cells in vitro, we have found that these cells formed tumours when they were transplanted in Fisher rats. We then confirmed that the tumour was of epithelial cell origin, and spleen cells were not necessary to induce the tumour. We report here the MDP-mediated in vivo transformation of FRTL-5 cells to cancer cells (FRTC). FRTC retained various characteristics of a well-differentiated thyroid carcinoma. FRTC responded to TSH stimulation to produce Tg and cAMP, and expressed Tg, TPO and TSH receptor mRNA. However, the growth of FRTC was independent of TSH.

*Received 19 April 1996*
*Revised 30 July 1996*
*Accepted 31 July 1996*

*Correspondence to:* M Iitaka

### MATERIALS AND METHODS

### Cell culture

FRTL-5 cells (donated by Dr Kohn, NIH, in 1987) were maintained in Ham's F-12 medium supplemented with 5% calf serum and six hormones (6H medium), as described previously (Ambesi-Impiombato et al, 1980). FRTL-5 cells were cultured in 6H medium in the presence or absence of 20 mg $l^{-1}$ MDP (Sigma, St Louis, USA) for 4 days at 37°C in 5% carbon dioxide. Cells were harvested after dispase digestion and $3 \times 10^5$ cells were injected intraperitoneally into Fisher rats. One month after the transplantation, disseminated peritoneal tumour was collected and digested with collagenase. Cells were then washed, suspended in the 6H medium and cultured in a plastic flask (Nunc, Roskilde, Denmark). As we found that the proliferation of these cells (FRTC) was independent of six hormones, Ham's F-12 medium supplemented only with 5% calf serum was used to maintain FRTC cells. Unless otherwise stated, FRTC cells of the third generation were used for the experiments.

**Figure 1** **A** Histological examination of peritoneal tumour developed in rats injected with MDP-treated FRTL-5 cells. Bar=50 μm. **B** Colloid-like structures in the tumour (arrow). Bar=100 μm

**Figure 2** **A** Immunoreactive Tg was detected in the colloid-like structures in the tumour. Bar=50 μm. **B** The thyroid follicles from the same rat stained with anti-Tg antibodies. Bar=50 μm

*© Cancer Research Campaign 1997*

Figure 3 Tumour formation in a Fisher rat after subcutaneous transplantation of FRTC cells

Figure 4 cAMP production in FRTL-5 (□) and FRTC (○) cells after TSH stimulation

### Tg measurement and staining

Culture supernatants from 1 × $10^5$ FRTL-5 and FRTC cells stimulated with various amounts of TSH for 24 h were collected. Tg in the supernatants was measured by enzyme-linked immunosorbent assay (ELISA) as described previously (Yanagisawa et al, 1986; Iitaka et al, 1991). Antibodies to rat Tg were prepared in the rabbit. Sections of paraffin-embedded tumour were stained with biotin-labelled anti-rat Tg antibodies and Vectastain ABC kit (Vector Laboratories, Burlingame, USA) according to the manufacturer's instructions. Control antibodies to rat lymphocyte surface antigens (OX6, OX8 and W3/25) were obtained from Harlan Sera-Lab (Crawley Down, UK).

### Responses to various agents

FRTL-5 cells (1 × $10^5$ cells per well) were cultured in 5H medium (without TSH) supplemented with 5% calf serum for 5 days before exposure to various stimuli. Recombinant insulin-like growth factor (IGF)-1 and mouse epidermal growth factor (EGF) were obtained from Toyobo (Osaka, Japan) and Sigma, respectively. Cells were stimulated with various stimuli for 24 h. Proliferation of FRTL-5 and FRTC cells was estimated by [$^3$H]thymidine (TdR, Amersham, Tokyo, Japan) uptake for 3 h as described previously (Iitaka et al, 1991). Cell proliferation was also estimated by the incorporation of crystal violet and photometric analysis as reported previously (Flick and Gifford, 1984). The cAMP content of supernatants after 3 h exposure to various stimuli was measured by radioimmunossay using commercially available kits (Yamasa, Tokyo, Japan). Incorporation of $^{131}$I was assessed as described previously (Ambesi-Impionbato et al, 1980).

Table 1 Effect of TSH on the proliferation of FRTC cells

| | TSH (mU l$^{-1}$) | | | | |
|---|---|---|---|---|---|
| | 0 | 0.01 | 0.1 | 1 | 10 |
| [$^3$H]TdR uptake (% of control) | 100[a] | 105±4 | 108±6 | 89±8 | 83±5 |

[a]The basal uptake was 6256 ± 462 c.p.m. (mean ± s.d. of quadruplicate cultures). The result is representative of three different experiments.

Table 2 The effect of various stimuli on the proliferation of FRTC cells

| Stimulus | [$^3$H]TdR uptake (% of control) |
|---|---|
| None | 100[a] |
| TSH (1 mU l$^{-1}$) | 87.2 ± 4.4 |
| IGF-1 (10 nmol l$^{-1}$) | 114.2 ± 4.9 |
| EGF (10 μ g l$^{-1}$) | 89.1 ± 2.9 |

[a]The basal uptake was 10 418±204 c.p.m. (mean ± s.d. of quadruplicate cultures). The result is representative of three different experiments.

### mRNA expression

Total RNA was extracted from FRTC tumours and FRTL-5 and FRTC cells as described previously (Chomczynski and Sacchi, 1987). When cells were used, they were seeded in 100-mm Petri dishes and cultured in 5H medium supplemented with 5% calf serum. Some cells were stimulated with 10 U l$^{-1}$ TSH for 24 h. In some experiments, total RNA was further purified with an oligo-dT cellulose column (Pharmacia, Uppsala, Sweden). Expression of various mRNAs was analysed by Northern blot hybridization using 10 μg of total RNA (for Tg, TPO, cyclophilin and β-actin) or 3 μg of poly-A RNA (for Tg, TPO, TSH receptor, c-*mvc*, c-*fos* and cyclophilin). Rat Tg cDNA (a gift from Dr DiLauro, European Molecular Biology Laboratory in 1988), rat TPO cDNA (Dr Rapoport, University of California, San Francisco, USA), rat TSH receptor cDNA (Dr Akamizu, Kyoto University, Japan; Dr Kohn, NIH, USA), rat cyclophilin cDNA (Dr Sutcliffe, Research Institute of Scripps Clinic, La Jolla, USA), v-*myc* and v-*fos* (Japanese Cancer Research Resources Bank) and β-actin cDNA (Nippon Gene, Tokyo, Japan) were used for hybridization. After the transfer of RNA, the membrane (Gene Screen Plus, Biotechnology Systems, Boston, USA) was incubated at 42°C for 15 min in 2 × standard saline citrate buffer (1 × SSC: 8.77 g sodium chloride, 4.41 g sodium citrate in 1 l of water), followed by the addition of a labelled probe (T7 Quick Prime kit, Pharmacia). The final washing

© Cancer Research Campaign 1997

**Figure 5** Effect of TSH on Tg production in FRTL-5 (□) and FRTC (○) cells

**Figure 6** Expression of Tg, TPO and β-actin mRNA in FRTL-5 and FRTC cells was with 0.1 × SSC at 60°C for 30 min for Tg, TPO, TSH receptor, cyclophilin and β-actin mRNA expression, and 1 × SSC at 60°C for 30 min for c-*myc* and c-*fos* mRNA. The membranes were then exposed to Fuji X-ray film at –70°C with an intensifying screen. Some membranes were also exposed to an imaging plate (Fuji Film, Tokyo, Japan) and analysed by BAS2000 (Fuji Film). Probes were removed from the membrane by boiling for 1 h in 0.1 × SSC, 1% sodium dodecyl sulphate (SDS). In some experiments, the same membranes were used successively for the detection of both specific and control (cyclophilin or β-actin) mRNAs.

All experiments were carried out at least twice using different batches of the cells of the same generation.

### RESULTS

### Transformation of FRTL-5 cells

Intraperitoneal transplantation of MDP-treated and untreated FRTL-5 cells into Fisher rats was performed on three different occasions. Disseminated intraperitoneal tumour formation with massive bloody ascites was observed in ten out of ten rats injected with MDP-treated FRTL-5 cells. Tumour formation was not observed in six out of six rats injected with untreated FRTL-5 cells. Histological examination revealed that the tumours consisted of atypical epithelial cells with irregular nuclei (Figure 1A). There was no histological difference in the tumours obtained on these three different occasions. There were colloid-like structures (Figure 1B) filled with Tg-positive material (Figure 2A). Figure 2B shows the thyroid follicles that were obtained from the same rat as shown in Figure 1B and stained with anti-Tg antibodies. Neither this colloid-like material nor real colloid in the rat thyroid was stained with antibodies to rat lymphocyte surface antigens, such as OX6, OX8 or W3/25 (data not shown). The tumour was digested and the cells were cultured in vitro. These cells (FRTC) also formed large encapsulated tumours in Fisher rats when transplanted subcutaneously (Figure 3). No tumour formation was observed when FRTC cells were transplanted in allogeneic Wistar rats. The doubling time of FRTC cells in vitro was 19 h, and that of FRTL-5 cells was 30 h. Karyotype analysis revealed that FRTC cells had 39–46 chromosomes with detectable rearrangements (mode 42.9), while our FRTL-5 cells had 37–40 chromosomes (mode 38.6). The original FRTL-5 cells have been reported to have 41–42 chromosomes (Ambesi-Impiombato et al, 1980).

### In vitro effect of MDP

FRTL-5 cells (1 × $10^4$ cells per well) were cultured in 5H or 6H medium for 4 days in the presence of various amounts of MDP, and [$^3$H]TdR incorporation for 3 h was examined at the end of the culture. In the absence of TSH, FRTL-5 cells did not proliferate significantly in the presence of MDP. In the presence of TSH, cells were about 80% confluent at the end of the culture. Even in the presence of TSH, however, there was no significant difference in [$^3$H]TdR incorporation between MDP-treated and untreated FRTL-5 cells (86–112% of the control in the presence of $10^{-5}$ to $10^{-1}$ g $l^{-1}$ MDP). Similarly, cAMP production was not markedly stimulated by MDP in FRTL-5 cells (85–114% of the control in the presence of $10^{-5}$ to $10^{-1}$ g $l^{-1}$ MDP). The morphology of FRTL-5 cells did not change even after long-term culture of up to 3 months in the presence of MDP.

### Response to various stimuli

FRTC cells produced cAMP in a dose-dependent manner after TSH stimulation (Figure 4). However, [$^3$H]TdR incorporation by FRTC cells was not markedly enhanced by TSH (Table 1). IGF-1 (10 nmol $l^{-1}$) slightly enhanced the proliferation of FRTC cells, although EGF (10μg $l^{-1}$) did not (Table 2). The basal uptake of $^{131}$I into FRTC cells was low (1.4±0.2% of total radioactivity in $10^5$ cells), and it was not enhanced by TSH stimulation (1.2±0.3%). Large amounts of TSH stimulated FRTC cells to produce Tg in vitro (Figure 5), although the response of Tg production to TSH stimulation in FRTC cells was poor compared with that in FRTL-5 cells.

### mRNA expression

Thyroid-specific mRNAs were expressed in FRTC cells in vivo as well as in vitro (Figure 6). Tg and TPO mRNAs were the same size in FRTC and FRTL-5 cells. TSH receptor mRNA was detected in FRTC cells in the presence and absence-of TSH, and

© Cancer Research Campaign 1997

**Figure 7** Expression of TSH receptor mRNA in FRTL-5 and FRTC cells in the presence or absence of TSH. TSH receptor mRNA in FRTL-5 cells decreased after TSH stimulation for 24 h, but increased in FRTC cells. The result is representative of three different experiments

**Figure 9** Expression of c-*myc* and cyclophilin mRNA in FRTC cells. Lanes 1,2 and 3 indicate c-*myc* and cyclophilin mRNA expression in FRTC cells cultured in the absence or presence of TSH, and in the presence of MDP and TSH, respectively. Lane 4 indicates c-*myc* and cyclophilin mRNA expression in the FRTC tumour tissue

**Figure 8** Expression of Tg, TSH receptor (TSH-R) and cyclophilin mRNA in tumours of FRTC of the third and the 12th generation

**Figure 10** Expression of c-*fos* and cyclophilin mRNA in FRTC cells. c-*fos* mRNA was expressed in FRTC cells cultured in the absence (lane 1) and in the presence (lane 2) of TSH, or in the tumour tissue (lane 3)

the size of the mRNA was the same in FRTL-5 cells. TSH receptor mRNA levels in FRTC cells increased after the TSH stimulation for 24 h, while levels decreased in FRTL-5 cells with TSH stimulation (Figure 7). The expression of Tg mRNA decreased in the tumour of FRTC of the 12th generation compared with that in the tumour of the third generation (Figure 8). On the other hand, there was no significant change in TSH receptor mRNA levels between these tumours (Figure 8).

It is of interest that c-*myc* and c-*fos* mRNA were consistently expressed in FRTC cells in vivo as well as in vitro. Expression of these proto-oncogenes was more prominent in vivo than in vitro (Figures 9 and 10). TSH did not enahnce the expression of c-*myc* or c-*fos* in FRTC cells (Figures 9 and 10). The expression of c-*myc* in FRTL-5 cells was not enhanced by MDP stimulation for 4 days (Figure 9).

### DISCUSSION

There have been several reports on the transformation of FRTL-5 cells with virus and/or active oncogenes (Fusco et al, 1981, 1985, 1987; Ferrentino et al, 1984; Berlingieri et al, 1990), chemical agents (Tramontano et al, 1986*a*; Sugawa et al, 1989) and the human TSH receptor cDNA (Derwahl et al, 1992). We have generated a malignantly transformed cell line, FRTC, by the transplantation of MDP-treated FRTL-5 cells into Fisher rats. All rats transplanted with MDP-treated FRTL-5 cells developed tumours. This result indicates that MDP may have a carcinogenic effect similar to ethyl methane sulphonate, a chemical mutagenic agent, which has been used to transform FRTL-5 cells (Tramontano et al, 1986a; Sugawa et al, 1989). However, MDP did not transform FRTL-5 cells in vitro, and tumours did not develop after the administration of MDP to Fisher rats (M Iitaka et al, unpublished data). MDP, also referred to as an adjuvant peptide, has been shown to stimulate T cells and macrophages (Ellouz et al, 1974; Lefrancier et al, 1977; Specter et al, 1977). There have been no previous reports concerning a possible carcinogenic effect of MDP on thyroid cells. We found that MDP had no stimulatory effect on FRTL-5 cells in vitro in terms of [$^3$H]TdR incorporation, cAMP production or c-*myc* expression. Since untreated FRTL-5 cells did not develop tumours, and the chromosomal heterogeneity and rearrangements in FRTC were evident as assessed by the karyotype analysis, MDP must have acted as a carcinogen or a tumour-promoting agent. Tramontano et al (1986) have found it necessary to use chemical mutagenesis to isolate TSH-independent clones from FRTL-5 cells because of the very low rate of spontaneous mutational events. However, Endo et al (1990) have successfully isolated a spontaneously transformed cell line from commercially available FRTL cells. This discrepancy may be a result of the difference of the cells. Similar to the previous report (Davies et al, 1987), our FRTL-5 cells also had the chromosomal heterogeneity. Huber et al (1990) have reported that there is extreme individual heterogeneity in human thyroid cells as well as FRTL-5 cells. They concluded that each thyroid cell has a highly variable growth programme, and non-transformed immortal cell lines in vitro may represent one end of a spectrum of individual growth potential

© Cancer Research Campaign 1997

among normal thyrocytes. In addition to such a high growth potential, some in vivo growth factor(s) in Fisher rats might have promoted the proliferation of transformed FRTL-5 cells in this study. Previous studies by Wollman (1963) have shown that thiouracil, a goitrogenic agent, promoted tumour formation of the thyroid gland in rats. Others have also reported that propylthiouracil, an anti-thyroid drug, greatly increased the incidence of thyroid tumour formation when rats were treated with *N*-bis(2-hydroxypropyl) nitrosamine (Kitahori et al, 1982) or the Kirsten murine sarcoma virus (Portella et al, 1989). As these goitrogenic agents induce the elevation of serum TSH levels, TSH may act as one of the tumour-promoting factors in those rats. Although the precise mechanism for the generation of FRTC cells with MDP treatment remains to be clarified, it is of interest that MDP, a ubiquitously present bacterial wall peptide, may act as a tumour-promoting or carcinogenic agent at least in some circumstances.

FRTC cells have several characteristics similar to FRTL-5 cells, such as Tg production and the expression of Tg, TPO and TSH receptor mRNA, although proliferation of FRTC cells was independent of TSH. Previous reports have shown that transformed FRTL-5 cells usually lose differentiation markers and TSH growth dependence (Fusco et al, 1981; Sugawa et al, 1989; Berlingieri et al, 1990). Wollman (1963) has also shown some tendency towards progressive dedifferentiation among the more differentiated thyroid tumours in rats. Differentiated human thyroid carcinomas, however, express Tg, TPO and TSH receptor mRNA to varying degrees (Brabant et al, 1991; Ohta et al, 1991). Similar to FRTL-5 cells and some of previously reported mutant clones (Tramontano et al, 1986*a*; Endo et al, 1990), FRTC cells produced cAMP and Tg in a dose-dependent manner when stimulated with TSH. Although this indicates that TSH may still continue to act as a stimulator of Tg production in FRTC cells, the elevated basal Tg levels and poor response of Tg to TSH stimulation also suggest a possibility that FRTC may be able to produce Tg, at least in part, independently of TSH. The discrepancy between TSH-independent proliferation and TSH-dependent increase in cAMP production in FRTC cells indicates that a signal transduction pathway(s) other than the TSH–cAMP-mediated pathway may primarily affect their growth. Although EGF may be a candidate growth factor for thyroid cells (Roger and Dumont, 1982; Westermark and Westermark, 1982), the proliferation of FRTC cells was not markedly enhanced by EGF stimulation.

The histological patterns of FRTC tumours exhibited considerable stability from generation to generation. However, the expression of Tg mRNA in the tumours of the 12th generation decreased compared with those of the third generation, indicating that there may be a tendency towards dedifferentition in FRTC. It is of interest that there was no change in the expression of TSH receptor mRNA between these tumours. This is compatible with the previous report that TSH receptor mRNA expression is retained much further along the pathway of transformation than the expression of function-related genes, such as Tg or TPO (Brabant et al, 1991).

It is of interest that the expression of TSH receptor mRNA increased after TSH stimulation in FRTC cells, but decreased in FRTL-5 cells, as reported previously (Akamizu et al, 1990). In normal and abnormal human thyroid cells obtained from patients with adenomas or papillary cancers, expression levels of TSH receptor mRNA have been reported to increase after TSH stimulation in vitro (Huber et al, 1991). Maenhaut et al (1992) reported that TSH mRNA levels in dog thyrocytes increased after the TSH stimulation for 20 h, but decreased afterwards. They also reported that neither 2-day forskolin stimulation nor 6 h treatment with TSH or forskolin in vitro had any influence on the TSH mRNA expression in human thyroid cells. The basis of this discrepancy, however, is unknown. In thyroid papillary or follicular carcinoma tissues, TSH receptor mRNA levels have been reported to vary from normal to markedly decreased (Brabant et al, 1991; Ohta et al, 1991). TSH receptor mRNA levels were high in benign thyroid tumours but not detectable in anaplastic cancers, indicating that the expression of TSH receptor mRNA levels may be dependent on the differentiation of thyroid cells.

Proto-oncogenes, such as c-*myc* and c-*fos*, were expressed in FRTC cells even in the absence of TSH stimulation. These proto-oncogenes were expressed at higher levels in vivo than in vitro. FRTL-5 cells have been reported to express these proto-oncogenes for a brief period when stimulated with TSH or dibutyryl cAMP (Colletta et al, 1986; Tramontano et al, 1986*b*), although others have shown that EGF, but not TSH, stimulates the expression of c-*fos* and c-*myc* mRNA in primary porcine thyroid cell cultures (Heldin and Westermark, 1988). In human thyroid cancer tissues, it has been reported that c-*myc* or c-*fos* mRNA is expressed at varying levels (Wyllie et al, 1989; Brabant et al, 1991). FRTC cells persistently express c-*myc* or c-*fos* mRNA in vitro without TSH stimulation. The persistent activation of c-*myc* or c-*fos* may induce unlimited proliferation of FRTC cells in the absence of TSH.

FRTC cells retain many characteristics of differentiated thyroid cancer cells and are transplantable to syngeneic Fisher rats. Although the mechanism of malignant transformation with MDP remains unknown, FRTC may be a useful cell line for the investigation of differentiated thyroid cancer.

### ACKNOWLEDGEMENTS

We gratefully acknowledge Ms Y Kuwahara and Ms T Suzuki for their skilful technical assistance. We also thank Dr Kohn, Dr Rapopport, Dr Di Lauro, Dr Sutcliffe and the Japanese Cancer Research Resources Bank for supplying us with FRTL-5 cells and cDNA probes. This work was supported in part by a grant-in-aid for scientific research (Nos. 05670880 and 06671051) from the Ministry of Education, Science and Culture.

### REFERENCES

Akamizu T, Ikuyama S, Saji M, Kosugi S, Kozak C, McBride OW and Kohn LD (1990) Cloning, chromosomal assignment, and regulation of the rat thyrotropin receptor: expression of the gene is regulated by thyrotropin, agents that increase cAMP levels, and thyroid autoantibodies. *Proc Natl Acad Sci USA* **87:** 5677–5681

Ambesi-Impiombato FS, Parks LAM and Coon HG (1990) Culture of hormone-dependent functional epithelial cells from rat thyroid. *Proc Natl Acad Sci USA* **77:** 3455–3459

Berlingieri MT, Akamizu T, Fusco A, Grieco M, Colletta G, Cirafici AM, Ikuyama S, Kohn LD and Vecchio G (1990) Thyrotropin receptor gene expression in oncogene-transfected rat thyroid cells: correlation between transformation, loss of thyrotropin-dependent growth, and loss of thyrotropin receptor gene expression. *Biochem Biophys Res Commun* **173:** 172–178

Brabant G, Maenhaut C, Kôhrle J, Scheumann G, Dralle H, Hoang-VU C, Hesch RD, Von Zur Mûhlen A, Vassart G and Dumont JE (1991) Human thyrotropin receptor gene: expression in thyroid tumors and correlation to markers of thyroid differentiation and dedifferentiation. *Mol Endocrinol* **82:** R7–R12

Chomczynski P and Sacchi N (1987) Single-step method of RNA isolation by acid guanidinium thiocyanate – phenol – chloroform extraction. *Anal Biochem* **162:** 156–159

© Cancer Research Campaign 1997

Colletta G, Cirafici AM and Vecchio G (1986) Induction of the c-fos oncogene by thyrotropic hormone in rat thyroid cells in culture. *Science* **233:** 458–460

Davies TF, Yang C and Platzer M (1987) Cloning the Fisher rat thyroid cell line (FRTL-5): variability in clonal growth and 3′,5′-cyclic adenosine monophosphate response to thyrotropin. *Endocrinology* **121:** 78–83

Derwahl M, Broecker M, Aeschimann S, Schatz H and Studer H (1992) Malignant transformation of rat thyroid cells transfected with the human TSH receptor cDNA. *Biochem Biophys Res Commun* **183:** 220–226

Ellouz F, Adams A, Ciorbaru R and Lederer E (1974) Minimal structural requirements for adjuvant activity of bacterial peptidoglycan derivatives. *Biochem Biophys Res Commun* **59:** 1317–1325

Endo T, Shimura H, Saito T and Onaya T (1990) Cloning of malignantly transformed rat thyroid (FRTL) cells with thyrotropin receptor and their growth inhibition by 3′,5′-cyclic adenosine monophosphate. *Endocrinology* **126:** 1492–1497

Ferrentino M, DI Fiore PP, Fusco A, Colletta G, Pinto A and Vecchio G (1984) Expression of the oncogene of the Kirsten murine sarcoma virus in differentiated rat thyroid epithelial cell lines. *J Gen Virol* **65:** 1955–1961

Flick DA and Gifford GE (1984) Comparison of *in vitro* cell cytotoxic assays for tumor necrosis factor. *J Immunol* **68:** 167–175

Fusco A, Pinto A, Saverio F, Ambesi-Impiombato FS, Vecchio G and Tsuchida N (1981) Transformation of rat thyroid epithelial cells by Kirsten murine sarcoma virus. *Int J Cancer* **28:** 655–662

Fusco A, Portella G, DI Fiore PP, Berlingieri MT, DI Lauro R, Schneider A and Vecchio G (1985) A mos oncogene containing retrovirus, myeloproliferative sarcoma virus, transforms rat thyroid epithelial cells and irreversibly blocks their differentiation pattern. *J Virol* **56:** 284–292

Fusco A, Berlingieri MT, DI Fiore PP, Portela G, Grieco M and Vecchio G (1987) One-and two-step transformations of rat thyroid epithelial cells by retroviral oncogenes. *Mol Cell Biol* **7:** 3365–3370

Heldin NE and Westermark B (1988) Epidermal growth factor, but not thyrotropin, stimulates the expression of c-fos and c-myc messenger ribonucleic acid in porcine thyroid follicle cells in primary culture. *Endocrinology* **122:** 1042–1046

Huber G, Derwahl M, Kaempf J, Peter HJ, Gerber H and Studer H (1990) Generation of intercellular heterogeneity of growth and function in cloned rat thyroid cells (FRTL-5). *Endocrinology* **126:** 1639–1645

Huber GK, Concepcion ES, Graves PN and Davies TF (1991) Positive regulation of human thyrotropin receptor mRNA by thyrotropin. *J Clin Endocrinol Metab* **72:** 1394–1396

Iitaka M, Fukasawa N, Yanagisawa M, Hase K, Miura S, Hara Y, Ishii J, Kawazu S and Komeda K (1991) Effect of cholera toxin on serum levels of thyrotropin and thyroid autoantibodies in BioBreeding/Tokyo (BB/TKY) rats. *J Clin Lab Immunol* **36:** 33–38

Kitahori Y, Hiasa Y, Konishi N, Enoki N, Shimoyama T and Miyashiro A (1984) Effect of propylthiouracil on the thyroid tumorigenesis induced by *N*-bis(2-hydroxypropyl)nitrosamine in rats. *Carcinogenesis* **5:** 657–660

Lefrancier P, Choay J and Lederman I (1977) Synthesis of *N*-acetyl-muramyl-L-alanyl-D-isoglutamine, an adjuvant of the immune response, and of some *N*-acetyl-muramyl-peptide analogs. *Int J Peptide Protein Res* **9:** 249–257

Maenhaut C, Brabant G, Vassart G and Dumont E (1992) In vitro and in vivo regulation of thyrotropin receptor mRNA levels in dog and human thyroid cells. *J Biol Chem* **267:** 3000–3007

Ohta K, Endo T and Onaya T (1991) The mRNA levels of thyrotropin receptor, thyroglobulin and thyroid peroxidase in neoplastic human thyroid tissues. *Biochem Biophys Res Commun* **174:** 1148–1153

Portella G, Ferulano G, Santoro M, Grieco M, Fusco A and Vecchio G (1989) The Kirsten murine sarcoma virus induces rat thyroid carcinomas in vivo. *Oncogene* **4:** 181–188

Roger PP and Dumont JE (1982) Epidermal growth factor controls the proliferation and expression of differentiation in canine thyroid cells in primary culture. *FEBS Lett* **144:** 209–212

Specter S, Friedman H and Chedid L (1977) Dissociation between the adjuvant vs mitogenic activity of a synthetic muramyl dipeptide for murine splenocytes. *Proc Soc Exp Biol Med* **155:** 349–352

Sugawa H, Mori T and Imura H (1989) Establishment of 8-azaguanine-resistant mutants from rat thyroid cell line FRTL-5. *Mol Cell Endocrinol* **62:** 319–326

Tramontano D, Rotella CM, Toccafondi R and Ambesi-Impionbato FS (1986*a*) Thyrotropin-independent mutant clones from FRTL5 rat thyroid cells: hormonal control mechanisms in differentiated cells. *Endocrinology* **118:** 862–868

Tramontano D, Chin WW, Moses AC and Ingbar SH (1986*b*) Thyrotropin and dibutyryl cyclic AMP increase levels of c-myc and c-fos mRNAs in cultured rat thyroid cells. *J Biol Chem* **261:** 3919–3922

Volpert EM and Prezyna AP (1977) Transplantable thyroid tumour in rats: iodoamino acid distribution in successive tumour generation. *Acta Endocrinol* **85:** 93–101

Wollman SH (1963) Production and properties of transplantable tumors of the thyroid gland in the Fisher rat. *Recent Progr Hormone Res* **19:** 579–618

Westermark K and Westermark B (1982) Mitogenic effect of epidermal growth factor on sheep thyroid cells in culture. *Exp Cell Res* **138:** 47–55

Wyllie FS., Lemoine NR, Williams ED and Wynford-Thomas D (1989) Structure and expression of nuclear oncogenes in multi-stage thyroid tumorigenesis. *Br J Cancer* **60:** 561–565

Yanagisawa M, Hara Y, Satoh K, Tanikawa T, Sakatsume Y, Katayama S, Kawazu S, Ishii J and Komeda K (1986) Spontaneous autoimmune thyroiditis in BioBreeding/Worcester rat. *Endocrinol Japan* **33:** 851–861

© Cancer Research Campaign 1997